# Biology, Pest Status, Microbiome and Control of Kudzu Bug (Hemiptera: Heteroptera: Plataspidae): A New Invasive Pest in the U.S.

**DOI:** 10.3390/ijms17091570

**Published:** 2016-09-16

**Authors:** Anirudh Dhammi, Jaap B. van Krestchmar, Loganathan Ponnusamy, Jack S. Bacheler, Dominic D. Reisig, Ames Herbert, Alejandro I. Del Pozo-Valdivia, R. Michael Roe

**Affiliations:** 1 Department of Entomology and Plant Pathology, North Carolina State University, Raleigh, NC 27695, USA; adhammi@ncsu.edu (A.D.); loganathan_ponnusamy@ncsu.edu (L.P.); jsbachel@ncsu.edu (J.S.B.); ddreisig@ncsu.edu (D.D.R.); aidelpoz@ncsu.edu (A.I.D.P.-V.); 2 Center for Integrated Pest Management, North Carolina State University, Raleigh, NC 27606, USA; jbkretsc@ncsu.edu; 3 Tidewater Agricultural Research & Extension Center, Suffolk, VA 23437, USA; herbert@exchange.vt.edu

**Keywords:** *Megacopta cribraria*, *Megacopta punctatissima*, *Candidatus* Ishikawaella capsulata, sampling, monitoring, cultural control, biological control

## Abstract

Soybean is an important food crop, and insect integrated pest management (IPM) is critical to the sustainability of this production system. In recent years, the introduction into the United States of the kudzu bug currently identified as *Megacopta cribraria* (F.), poses a threat to soybean production. The kudzu bug was first discovered in the state of Georgia, U.S. in 2009 and since then has spread to most of the southeastern states. Because it was not found in the North American subcontinent before this time, much of our knowledge of this insect comes from research done in its native habitat. However, since the U.S. introduction, studies have been undertaken to improve our understanding of the kudzu bug basic biology, microbiome, migration patterns, host selection and management in its expanding new range. Researchers are not only looking at developing IPM strategies for the kudzu bug in soybean, but also at its unique relationship with symbiotic bacteria. Adult females deposit bacterial packets with their eggs, and the neonates feed on these packets to acquire the bacteria, *Candidatus* Ishikawaella capsulata. The kudzu bug should be an informative model to study the co-evolution of insect function and behavior with that of a single bacteria species. We review kudzu bug trapping and survey methods, the development of bioassays for insecticide susceptibility, insecticide efficacy, host preferences, impact of the pest on urban environments, population expansion, and the occurrence of natural enemies. The identity of the kudzu bug in the U.S. is not clear. We propose that the kudzu bug currently accepted as *M. cribraria* in the U.S. is actually *Megacopta* *punctatissima*, with more work needed to confirm this hypothesis.

## 1. Introduction

**Figure 1:**
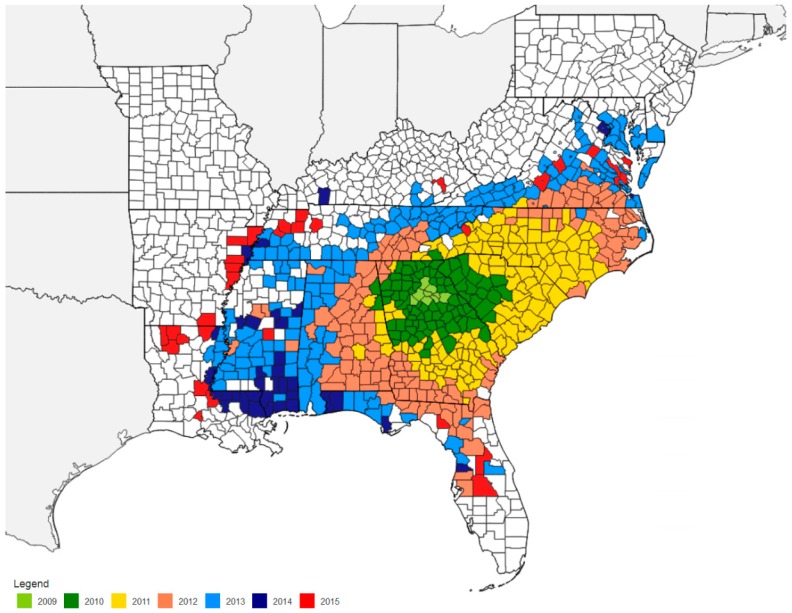
Distribution of the kudzu bug *Megacopta cribraria* in the southeast U.S. from 2009–2015. Map is compiled by Wayne A. Gardner [6], University of Georgia (available at http://www.kudzubug.org/) (accessed on 20 November 2015).

Kudzu bug, *Megacopta cribraria* (F.) (Hemiptera: Heteroptera: Plataspidae), is native to the Old World, i.e., the Eastern hemisphere continents of Asia and Australia, where it is commonly known as bean plataspid, lablab bug and globular stink bug [1]. Prior to 2009, sightings and studies of plataspid species were limited to Pakistan, India, Sri Lanka, China, Korea, Japan, Myanmar, Thailand, Vietnam, Macao, Taiwan, Malaysia, Indonesia, Australia, and New Caledonia [1]. No kudzu bug or for that matter any member of the insect family, Plataspidae, had been described in the continental U.S. before 2009 [2]. In October 2009, large numbers of the kudzu bug were discovered on the walls of homes near kudzu, *Pueraria montana* Lour. (Merr.) variety *lobata* (Willd.) Ohwi, in nine counties in northeast Georgia, U.S. By August 2010, the insects were found in 48 counties in northeast Georgia and 13 counties in northwest South Carolina [1]. Two years after its discovery, populations of *M. cribraria* had spread to most of Georgia and South Carolina and much of North Carolina [3], with additional sightings in Virginia and Alabama [4,5]. Presently, the kudzu bug has spread into all South Carolina counties; most counties in Georgia and North Carolina; has been found in Alabama, in most soybean growing counties in Virginia, Florida, Tennessee and Mississippi; and in parts of Arkansas and Louisiana (Figure 1) [6] and continues to expand each year [6].

Soybeans are an economically important U.S. crop. Since 2008, annual soybean production in the U.S. exceeded 34 million hectares [7]. In 2014, soybean covered 708,199.87 hectares in North Carolina, 267,092.52 hectares in Virginia, 663,684.45 hectares in Tennessee, 182,108.54 hectares in South Carolina, and 121,405.69 hectares in Georgia [8]; the sustainability of this crop is critical to the economies in the mid-Atlantic and southeast regions and the U.S. as a whole. The kudzu bug as a pest of soybean can cause a substantial reduction in yield [9,10,11] to be discussed in more detail later (Section 5). The adults are also considered by some to deleteriously impact urban environments. Kudzu bugs in the fall can move to urban structures especially outside walls, where the bugs can defecate producing brown spots on walls or serve as a general nuisance to residents [2]. However, their preference is to overwinter elsewhere. They potentially also can cause direct human damage to skin and eyes from their defensive chemicals [12]; the composition of these chemicals has not been characterized.

Up until the time of the kudzu bug introduction, there was virtually no research interest in this insect in the U.S. Since then, researchers have investigated numerous aspects of this invasive insect. This paper is aimed at reviewing the historic and recent, largely U.S., research into its identification, biology and life history, including endosymbionts and its economy, host preferences and management (Table 1).

## 2. Identification of the Kudzu Bug and Its Bacterial Symbionts in the U.S.

The kudzu bug was first identified in the U.S. as the bean plataspid, *Megacopta cribraria* (F.), using morphological characters by Eger et al. [1]. In subsequent work, genomic DNA was obtained from three specimens collected from northeast Georgia and the cytochrome oxidase subunit 1 (CO1) was amplified and sequenced. On the basis of these insect samples, a single maternal line was suggested since the nucleotide sequences were identical between insects. The sequences were aligned against GenBank reference sequences and found to be 98.6% identical to *M. cribraria* [23]. Primers (16SA1 and 16SB1) specific to the bacterial 16s rRNA gene were used to amplify DNA from the putative *Candidatus* Ishikawaella capsulata (a γ proteobacteria) expected to be found in *M. cribraria* (discussed in more detail later in this section). The amplified 16S rDNA was sequenced, and the alignment from the three specimens examined was 100% homologous with *Candidatus* Ishikiwaella capsulata, providing further evidence at least that the insects found in the U.S. contained the same symbionts [23].

Jenkins and Eaton [24] working under the assumption that the kudzu bug in the U.S. was *M. cribraria*, examined a number of maternal lineages of the insect by isolating DNA from 83 individuals collected in GA and SC. Using appropriate primers, a 2336 bp mitochondrial DNA fragment was isolated and sequenced from each. Alignments showed the sequences were the same for the 83 insects examined. For five individuals collected from four GA counties, Jenkins and Eaton [24] sequenced the entire 15,647 bp mitochondrial genome. Alignments showed the sequences to be the same. This evidence of a single maternal haplotype further supported the conclusion that a single maternal line designated GA1 had been introduced in 2009.

Jenkins and Eaton [24] also amplified and sequenced two genes, 16S rRNA and *groEL* chaperone, from the bacterial genome of the *Candidatus* Ishikawaella capsulata collected from insects in the U.S. Additionally, they amplified a gene coding the outer surface protein gene, *wsp*, from *Wolbachia*. Alignment against GenBank database sequences showed that: (i) the sequences for the 16S rRNA gene were identical to GenBank sequences for *Candidatus* Ishikawaella capsulata from Japanese *M. punctatissima*; (ii) the sequences for the *groEL* chaperone gene were 99% identical to GenBank sequences for *Candidatus* Ishikawaella capsulata from Japanese *M. punctatissima* and *M. cribraria*; and (iii) sequences for the *wsp* gene were 100% identical to GenBank sequences for *Wolbachia* from Japanese *M. punctatissima*. When DNA from a museum specimen, originally collected from the Japanese Ryuku Islands and identified as *Coptosoma cribraria* (Hemiptera: Plataspidae) in 1952 was analyzed, the *Wolbachia* *wsp* sequences were homologous to those in the GA1 individuals and the Japanese *M. punctatissima* GenBank sequences. The 1952 museum specimen had recently been re-identified as a specimen of *Megacopta cribraria* [1]. These reports raise some questions about the species identification of *M. cribraria* in the U.S.

Probably the most definitive work on the species identification of the kudzu bug so far in the U.S. was reported by Hosokawa et al. [25]. They examined the 8.7 kb mitochrondrial DNA in different native populations of *M. cribraria*, *M. punctatissima* and what was described as a morphological intermediate, each found in different geographical areas in East Asia. Phylogeographic genetic analyses identified eight distinct clades (A–H) each specific to a different geographical area and within each clade either a single species or in the case of one clade a morphological intermediate. When they conducted the same analysis with *M. cribraria* collected from its native range in the U.S., these U.S. insects were grouped into clade “E”, which geographically and by morphological classification belong to a population of *M. punctatissima* found in the Kyushu region*.* Their study concluded that what is called *Megacopta cribraria* in the U.S. is most similar to *M. punctatissima*.

This potential mis-identification of the U.S. kudzu bug is supported by another work. *M. punctatissima* is a known pest of soybean and pea, *Pisum sativum* L., in Japan, with only occasional reports of *M. cribraria* in soybean [44]. The primary host plant for *M. punctatissima* in Japan was identified as the leguminous vine, *Pueraria lobata*, while the primary host of *M. cribraria* in Japan was identified as *P. montana*. The bacterial microbiome of these two species is thought to play a role in conferring the ability to feed on soybean. Symbionts of these two insects are harbored in the insect’s posterior midgut and are vertically transferred in capsules during oviposition. The capsule of symbionts are deposited under the egg mass on the host plant surface. Neonates acquire the symbionts by feeding on the capsules. When the symbiont capsules of these two species are experimentally switched and these two species are reared on soybean, the egg hatch rate from *M. punctatissima* females which feed normally on soybeans drops to about 55% while that for *M. cribraria* females that do not feed typically on soybeans increases to approximately 40% [44].

In summary, the following possibilities exist: (i) *M. cribraria*’s ability to feed on soybean in the U.S. is possible because it has the same symbionts that allow *M. punctatissima* to feed on soybean in Asia; (ii) the symbionts in *M. cribraria* have evolved in the U.S. since 2009 from those in Asia to permit feeding on soybean in the U.S.; (iii) *M. cribraria* in the U.S. has been misidentified, and this insect is actually *M. punctatissima*. There also appears based on the study of Hosokawa et al. [25] that intermediates are possible between *M. cribraria* and *M. punctatissima*, which makes identification more complicated; or (iv) *M. cribraria* and *M. punctatissima* are the same species and the difference between their morphology, host range and other differences are just the manifestation of differences in endosymboints. At present, the issue of whether *M. cribraria* and *M. punctatissima* are distinct species cannot be definitively resolved by the current published literature. Since the species present in the Southeast U.S. is currently recognized by most U.S. investigators as *M. cribraria* [12], it will be referenced as such for the remainder of this review. However, more work is needed comparing the U.S. insects and their symbionts to those from their geographical area of origin to better understand what insect has been introduced into the U.S.

## 3. Kudzu Bug Host Plants Include Soybean

If the kudzu bug were only a pest of kudzu, *Pueraria montana* Lour (Merr.) variety *lobate* (Willd.) Ohwi, it would probably be considered in the U.S. as a beneficial insect since it can reduce kudzu biomass [3]. However, the preferred hosts of the kudzu bug include both wild and cultivated species of legumes, including soybean [1]. Wild legume species, primarily kudzu, enhanced the pest status of *M. cribraria* in soybean by providing a site for oviposition on kudzu in the spring for adults that survived the winter. Soybean would not have been planted at this time and/or not grown enough to support the overwintered populations. However, recent studies have confirmed that adult kudzu bugs emerging from overwintering sites can also colonize early-planted soybean [14].

It appears the insect stage most successful for overwintering is the adult stage [12,22]. Whether the insect has an adult diapause needs further study. When adults are transferred from the field in the winter to the laboratory at room temperature, they become active in minutes and within a week begin to oviposit eggs [14]. It is not clear if this is diapause or quiescence.

The growth and development of the insect on kudzu in the spring produces adults which disperse to soybean in the summer [26]. Other leguminous wild host species including wisterias, vetches and lespedezas [1,3,5], and several tree species [3] have been reported as wild hosts. However, it is unclear whether these plants only provide temporary feeding or insects can complete their life cycle. Some of the hosts were reported from non-U.S. locations [1] and others are based on small cage studies and may not represent what happens in the field [3]. Although more host plants may exist in the U.S. that will support completion of the full life cycle, the production of a complete generation has only been documented on kudzu and soybean [3]. Additional field leguminous hosts include pinto bean, lima bean, winter pea, and black-eyed pea [27,28,49]. The migration of *M. cribraria* adults from kudzu to soybean has been reported in the southeastern U.S. by several investigators [3,4,13]. Movement from kudzu and other wild legume hosts occurs from July to August [4,13].

In a field-choice test in Georgia using soybean, kudzu and other forest legume species, Zhang et al. [3] observed an oviposition preference for kudzu (528.8 eggs/plant) > soybean (320.0 eggs/plant) > *Lespedeza hirta* (122.2 eggs/plant) > *Lespedeza cuneata* (108.4 eggs/plant) > *Wisteria frutescens* (18.8 eggs/plant). This report was not an accurate representation of ovipositional preference, however, since small plots were created within a kudzu patch, skewing preference toward kudzu. In addition, adults could easily crawl from the kudzu into the small plots rather than their common method of flight dispersal. Furthermore, kudzu used in the experiment had to be cleared of *M. cribraria* eggs before it was placed into the plots. If an aggregation pheromone is involved in adult choice for feeding or oviposition, results would also likely to be skewed toward kudzu. Nonetheless, they found that of kudzu, soybean and 11 other tested forest legume species, *M. cribraria* completed development on only kudzu and soybean [3]. Similar results were also found by Huskisson et al. [50] where they showed that the kudzu bug preferred soybean over lima beans.

Medal et al. [27] showed that kudzu bugs could also complete development on pigeon pea, black-eye pea, lima beans and pinto beans. In this study, the number of adults produced on pigeon pea was higher (75 from five egg masses) as compared to black-eye pea (27 from three egg masses), lima beans (5 from one egg mass) and pinto beans (1 from one egg mass). The Zhang et al. [3] and the Medal et al. [27] studies mostly examined different host plants. However, one exception was the black-eye pea where results were inconsistent between studies; Zhang et al. [3] showed no adult development on black-eye pea while Medal et al. [27] had opposite results. Another greenhouse and field study conducted from 2012 to 2013 concluded that soybean, edamame and pigeon pea were suitable for kudzu bug development [28]. A later study conducted by Golec et al. [49] confirmed that kudzu bug can oviposit and develop not only in soybean, but also in mung bean and lima bean. Kudzu became an invasive weed in the U.S. after being exhibited as a perennial ornamental Chinese vine at the 1876 Philadelphia Centennial Exposition [3]. In the U.S., over 2.8 million ha now have become infested with kudzu with a range expansion rate of over 50,000 ha/year. Although host preference results with soybean, kudzu and 11 other species of forest legumes tested in Georgia suggested a limited host range for the kudzu bug, Zhang et al. [3] suggested over 100 protected legume species in North American forests (but proximal to soybean) could support oviposition, development and overwintering of the kudzu bug. The acquisition of bacterial symbionts like Ishikawaella [47] and further studies of the kudzu bug microbiome are needed to understand impact of associated microorganisms on host selection, host transitions, survival, development and reproduction.

The prospect of yet unidentified wild hosts supporting kudzu bug development in soybean-growing areas of the U.S. is alarming, but equally alarming is the potential for *M. cribraria* to attack non-leguminous U.S. crops. DNA analysis of the exon of the chloroplast trnL gene in the gut of kudzu bugs in the U.S. indicate they feed on many angiosperms and conifers in addition to kudzu and legumes [48]. These findings infer that the kudzu bug might have a broader impact in the future than initially thought. In China, the bug is reported to be a pest of peach, *Amygdalus persica* Linn.; plums, *Prunus* spp.; and other tree fruits [3]. Srinivasaperumal et al. [29] reported that *M. cribraria* also was not only a serious pest on hummingbird tree/scarlet wisteria, *Sesbania grandiflora*, and the firecracker flower, *Crosandra undulaefolia*, but also occurred sporadically on upland cotton, *Gossypium hirsutum*. They provided detailed life history studies of the insect reared in the laboratory on these same plants and found that *M. cribraria* feeding on *S. grandiflora* manifested a higher fecundity rate and survival than on the other plants examined. There so far is no evidence in the U.S. that the kudzu bug will feed on cotton or on other non-legume hosts. In recent experiments at North Carolina State University (Raleigh, NC, USA), eggs produced from field collected kudzu bug adults from the local area were transferred to cotton (PHY425RF) plants and allowed to hatch in the laboratory. The neonates were provided no other food source, and the insects died of starvation without any observable feeding on the cotton plant. When cotton (PHY425RF) plants grown in pots were placed into the middle of a large kudzu growing area (50 m^2^), which were heavily infested with kudzu bugs, the insects ignored the cotton and preferentially fed on the kudzu. Therefore, it is difficult to reconcile the observations of Srinivasaperumal et al. [29] showing detailed developmental studies of the kudzu bug on cotton plants in laboratory studies in India with observations to the contrary in the field and lab in the U.S., with the lack of reports showing kudzu bug feeding on cotton plants in the U.S., and with recent reports of the same from India. Whether a shift in the insect’s microbiome or its genetics has occurred or there was a misidentification of the kudzu bug by Srinivasaperumal et al. [29], it is impossible to determine at this juncture. Additionally, the genetic diversity of the insects in the U.S. is likely smaller than in its native range outside of the U.S. which may have affected movement to other hosts. More information about feeding and development on different hosts is discussed elsewhere (Section 4 and Section 8) in this review.

## 4. Kudzu Bug Biology

**Figure 2:**
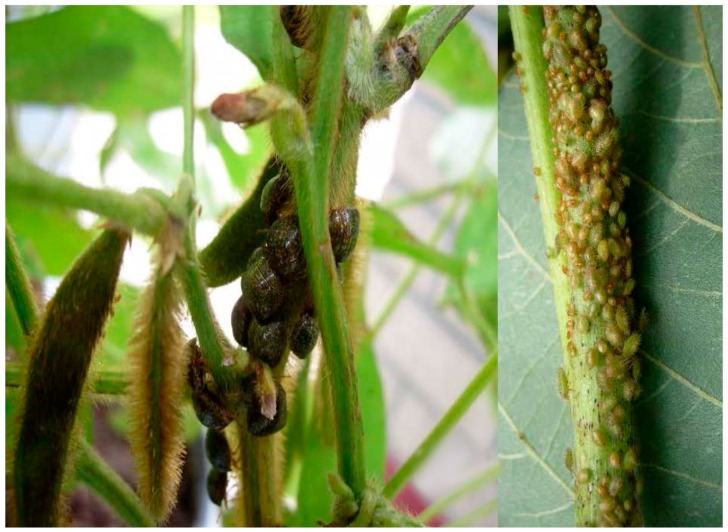
Kudzu bug, *Megacopta cribraria,* adults (**left**) and immatures (**right**) on U.S. soybean. The high insect density demonstrates the high potential for host damage.

Newly-molted adults of the kudzu bug are whitish in appearance, while the coloration of hardened adults is described as brown speckles on olive green [13] or mottled brown (Figure 2). Apart from slight differences in average size, females and males can be distinguished on the basis of their terminal sternites, V-shaped and characterized by a distinct suture in females and rounded in males [3].

**Figure 3:**
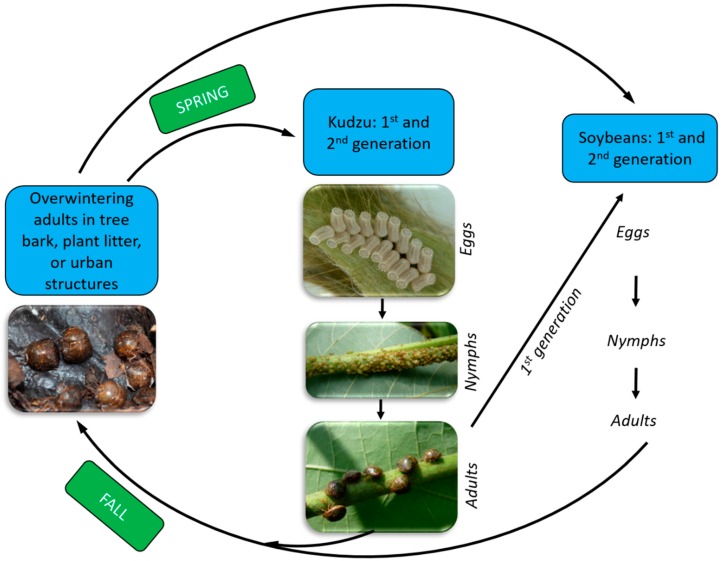
Typical life cycle and development stages of the kudzu bug, *Megacopta cribraria* (F.) (Hemiptera: Plataspidae), in the southeast United States.

Kudzu bug eggs are oval-shaped (0.9 mm long and 0.5 mm wide) and white soon after oviposition on leaves and off-white or pink thereafter [3]. On kudzu, the eggs are most commonly deposited in rows or groups of an average of 15.6 eggs per mass on leaf sheaths of younger, actively-growing vine tips (Figure 3). The bugs overwinter as adults near kudzu or soybean in the U.S. and can be found in plant litter, under tree bark and rocks, and in the walls and attics of homes and other buildings [3,13,22]. The adults are strong fliers. In the spring, prior to soybean planting, the overwintered adults fly to wild legume hosts such as kudzu and wisteria to feed and oviposit, producing the first field generation [13,15] (Figure 3), although a portion of the overwintering population colonizes early-planted soybean plants directly [14].

To date, Virginia is the northern most state in the U.S. where kudzu bug is established on a wide scale. State wide surveys of soybean fields from 2013 to 2015 have determined that they are presently found in 90 percent of the Virginia counties growing soybean from the southern border with North Carolina, to Maryland in the North, and as far west as north-central Louisiana and eastern Arkansas. To evaluate adult overwintering activity and movement in Virginia, a series of adult sticky traps was deployed in 2014 and 2015. The construction of these traps is discussed in section 10 of this review. Traps were placed in 51 counties scattered throughout Virginia and monitored from early March when temperatures first began to warm until early May. Operators checked traps weekly, changed the sticky surface, and recorded the date of the first time an adult kudzu bug was collected. Trap results showed that adults were captured from 18 counties, mostly in the southeast and south-central regions, beginning as early as 31 March through 7 May. These adults most likely had overwintered locally since the migration time from further south of the collection sites was short and their preferred host plants (kudzu and early planted soybean) were not available before the time of capture.

Golec et al. [16] recently studied over wintering kudzu bugs and found that 15% of the females were mated before overwintering. In that study, adult males and females were collected from Auburn, Alabama from September 2013 through March 2014. Females were found to not only be mated before overwintering, but they were also capable of storing sperm for up to seven months. All overwintering males had sperm in their testis indicating that both male and females can undergo reproductive dormancy. As discussed before, more work is needed to determine whether this is quiescence or diapause. The ratio of females to males increased continuously in overwintering populations, migration of the first field-generation adults from kudzu to soybean occurred over several weeks from July to August (Figure 3).

Numerous studies in the native range of *M. cribraria* revealed that adult females oviposit 26–274 eggs; that development from the egg through five stadia was 24–56 days; and that adult longevity ranged from 23 to 77 days with variation attributed to temperature, location and other factors like nutrition [1]. Del Pozo-Valdivia and Reisig [14] reported that on average, females laid 18 eggs with a developmental time that varied from 7 to 9 days. Nymphs required an average of 7.8, 8.5, 8.2, 7.8, and 6.6 days to complete the first to fifth stadia, respectively, at 28 °C, 60% relative humidity (RH) and 14:10 light:dark (L:D) cycle. In an additional study, development from egg to adult was 6–8 weeks depending on temperature [15]. Shi et al. [17] investigated the effect of five constant temperatures (17–33 °C) on development, survival and fecundity of the kudzu bug. This study concluded that the developmental time reduces significantly with an increase in temperature. Kudzu bugs had the shortest development time (38.54 days) at 29 °C and the longest at 17 °C (114.81 days). However, at 33 °C, they were not able to complete full development. Females had the longest oviposition period of 35.33 days and the highest fecundity rate of 159.67 eggs/female at 25 °C. These observations suggest temperature was a determining factor in population dynamics of the kudzu bug as would be expected for any ectotherm.

One to three generations of *M. cribraria* occurred per year in China and Japan [1]. In an Asian population of *M. cribraria*, Srinivasperumal et al. [29] found that at 27 °C and 14:10 (L:D), the egg stage was 4 days; the nymphal period averaged from 20 to 25 days (five stadia), and adults lived on average from 2.5 to 4 days on different plant hosts, *S. grandiflora*, *C. undulaefolia* and *G. hirsutum* (though our review suggests that this may have been a different plastipid species). Male average weight was greater than that of females on *S. grandiflora* and *G. hirsutum* and the reverse occurred for *C. undulaefolia*. In kudzu in Georgia, Zhang et al. [3] observed two and a possible partial third generation per year of *M. cribraria* with significant year-to-year variation. In 2010, oviposition peaks occurred in April and late July to early August; the following year, oviposition peaks occurred in April and June.

## 5. Soybean Damage from Kudzu Bugs

Kudzu bugs have piercing-sucking mouthparts. They are putative phloem feeders that reduce soybean yield by feeding on stems, leaf petioles (Figure 2) and foliage [2,51] and by diverting nutrients and moisture from plant vegetative and reproductive growth [13]. Kudzu bug are generally found forming aggregations on the main stem of soybean plants [18,19]. When infesting soybean fields, kudzu bugs have a tendency to occupy the edges of fields first, and then move from the field edges to the interior of fields later in the season, indicating that their in-field spatial distribution changes through time [20]. Studies in southern and central China demonstrated that heavy feeding caused some defoliation, and additional interference of photosynthesis occurred from sooty mold growth on *M. cribraria* excretions [3]. Yield reduction cage studies in South Carolina, U.S. [9] and field studies in Georgia, U.S. [10] revealed significant reductions in seed size and seed weight (but not pods per plant), seeds per pod, number of seeds per plant, and protein and oil content. Yield losses in soybean due to kudzu bug damage have been reported in the range of 1%–50% [1]. At one site in South Carolina, yield reduction for untreated soybean was 60% [9], while average yield losses by this pest in 19 replicated efficacy and threshold tests in Georgia and South Carolina in 2010 and 2011 were 18% (excluding its impact on seed quality) [12]. Finally, a series of field experiments demonstrated a yield loss of 51%, 28%, 16% and 6% in April, May, June and July planted soybean, respectively in Georgia [10]; and a range of 30% to 10% soybean yield reduction in South and North Carolina, respectively, when insecticide treated plots were compared against untreated plots [11].

## 6. The Identity of Plataspid Endosymbionts

Fukatsu and Hosokawa [45] investigated the endosymbionts associated with the Japanese common plataspid, *Megacopta punctatissima*. They concluded that one species of bacteria dominated the endosymbionts; this was based on a RFLP analysis of 30 eubacterial 16S rDNA clones amplified from DNA isolated from endosymbiont capsules produce by the females at the time of oviposition [45]. Sequencing and phylogenetic analysis of the 16S rDNA clones placed the bacteria in the γ-subdivision of Proteobacteria [45]. Hosokawa et al. [46] extracted DNA from female posterior midguts of four species of plataspids in Japan: *M. cribraria*, *M. punctatissima*, *Brachyplatys subaeneus*, and *Coptosoma parvipictum.* From this DNA, they PCR amplified the bacterial 16S rRNAgene, cloned the fragments, and genotyped the clones using RFLP. On the basis of RFLP DNA profiles, they concluded that a single and specific bacterial symbiont species was associated with each of the plataspid species examined [46]. Clones of the bacterial 16S rRNA gene from the female midgut DNA samples were sequenced, the sequences were aligned using ClustalW (European Bioinformatics Institute, Hinxton, Cambridge, UK), and phylogenetic trees estimated by using three methods, i.e., Bayesian, maximum likelihood and maximum parsimony. Hosokawa et al. [46] concluded that the gut endosymbionts of the plataspid examined form a distinct group within the γ-Proteobacteria and proposed the designation “*Candidatus* Ishikawaella capsulata”.

## 7. The Role of Endosymbionts in Development of Plataspids

As mentioned earlier in this review, symbionts of the kudzu bug are harbored in the insect’s posterior midgut and are vertically transferred in capsules during oviposition. The capsules of symbionts are deposited under the egg mass on the host plant surface. Neonates acquire the symbionts by feeding on the capsules. Fukatsu and Hosokawa [45] examined the developmental effects of depriving neonate *M. punctatissima* of viable endosymbionts by heat-treating the capsules normally associated with egg masses. They found that compared to a control group (of viable endosymbiont packets) for *M. punctatissima* nymphs, the nymphs associated with heat-treated endosymbiont capsules showed delayed development (from the first to the last stadium), reduced body mass, and abnormal coloration. Hosokawa et al. [46] examined the effect of removing endosymbiont capsules from the egg masses of four species of plataspids in Japan. They found significantly reduced adult emergence, reduced body size, and reduced pigmentation in *M. cribraria*, *M. punctatissima*, *B. subaeneus*, and *C. parvipictum*. They also found a significantly prolonged development time for *M. cribraria* and *M. punctatissima* nymphs that hatched from egg masses from which the endosymbiont capsules had been removed. On the basis of these experiments, Hosokawa et al. [46] concluded that the gut bacteria function as obligate mutualistic symbionts. In a related phylogenetic analysis, the sister group to the plataspid extracellular endosymbionts was the endocellular symbiont of aphids, *Buchnera aphidicola* [46]*.* In addition to plataspid stinkbugs and aphids, the development of many other plant-feeding species of Hemiptera is dependent on nutritional endosymbionts [45,46,52].

## 8. The Role of Endosymbionts in the Crop-Pest Status of Plataspids

The importance of symbiont capsules to the ability of *M. punctatissima* and *M. cribraria* to feed on soybean [44] has already been discussed earlier in this review (Section 2). Hosokawa et al. [44] concluded that the symbiont genotype confers crop-pest status to plataspid stinkbugs rather than the stinkbug species’ own genotype.

In the U.S., *M. cribraria* is an invasive species that is not a pest of soybean in Japan; however, in the U.S., the insect is a pest of soybean. The evolution of the endosymbiont, Ishikawaella, was studied by Brown et al. [47] in an effort to understand this discrepancy. The objective was to determine whether the Ishikawaella genome had changed before or after the U.S. introduction. A genomic analysis (allele frequency) of Ishikawaella from its first days of invasion into soybean in 2009 at 23 locations across the U.S. was conducted in 2011 and compared with the pest-conferring Ishikawaella genome from Japan. This study also showed that the U.S. Ishikawaella genome is identical to the Japanese post-conferring Ishikawaella genome in gene order and orientation. The U.S. Ishikawaella genome is 46 bp longer than its Japanese counterpart with only 47 differences, which included six indels and 41 substitutions. Only one gene was involved in having a nutritional benefit. Study also showed there were no fixed substitutions in U.S. populations of the symbiont. These results suggest that *M. cribraria* was capable of feeding on soybean at the time of its introduction into the U.S. This study, based on allele frequency analysis, also found differential selection of nutritional provisioning genes in kudzu and soybean between 2009 and 2011 further highlighting the role of this symbiont in host selection and its expansion to new hosts.

## 9. Characterization of the Bacterial Diversity of the Kudzu Bug by Denaturing Gradient Gel Electrophoresis (DGGE)

To assess the potential diversity of bacteria in the symbiont capsules and midguts of the kudzu bug, denaturing gradient gel electrophoresis (DGGE) was conducted of the amplified bacterial 16S rRNA gene by our research group. Female kudzu bug adults were collected from kudzu in Raleigh, NC, U.S. and allowed to oviposit their eggs and deposit packets in containers in the laboratory. From these samples, packets were separated from the eggs with sterile forceps. Before midgut dissections, female adults were immersed in a 95% ethanol solution for 30 s, washed three times with sterile distilled water followed by washing three times for 30 s each with 0.5% sodium hypochlorite, and then washing five times with sterile distilled water. Total DNA was extracted separately from three packets and three midguts as described by Ponnusamy et al. [53] and DNA purified with the WIZARD DNA Clean Up System (Promega, Madison, WI, USA). Purified DNA was subsequently used as a template to amplify the variable V3 region of the 16S rRNA using universal bacterial primers F357-GC (5′-GC-clamp+CCTACGGGAGGCAGCAG-3′) and 518R (5′-ATTACCGCGGCTGCTGG-3′) [54]. DGGE [55] was used to assess microbial diversity using the Dcode™ universal mutation detection system (Bio-Rad, Hercules, CA, USA) with linear chemical gradient of 45%–55% as described by Ponnusamy et al. [54].

**Figure 4:**
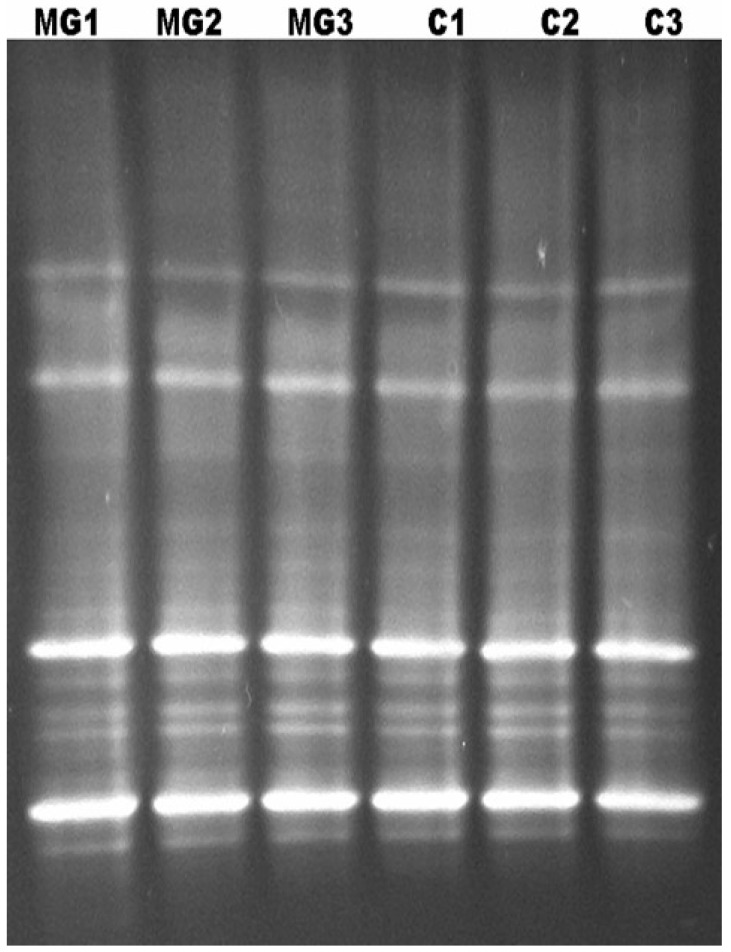
Denaturing gradient gel electrophoresis (DGGE) of PCR products from the V3 region of 16S rDNA for bacteria found in the midgut and capsule of kudzu bugs collected in Raleigh, NC. MG, midgut; C, capsules; 1–3, replicates.

The DGGE results demonstrated that the bacterial capsules contain more than one bacterial species (Figure 4) in contrast with earlier studies suggesting a single species predominates as the endosymbiont critical to kudzu bug development [45,46]. The results also show that the gut and capsule present similar DGGE patterns (Figure 4), supporting earlier work that bacteria in the gut are transmitted by means of the capsule to the next generation [45]. In this DGGE analysis, each band is likely to represent at least one unique bacterial species or phylotype, but in some circumstances single bacteria can represent multiple bands. Some PCR amplicons can produce more than one band when analyzed by DGGE, and a single bacterial species may produce more than one band because of heterogeneity of small subunit rRNA gene operons in a single bacterial strain. This can lead to an overestimate of bacterial species diversity [56,57,58]. Although this approach is a cost effective analysis to gauge the potential for bacteria diversity, high throughput sequencing has become the state-of-the-art technique for the analysis of microbial populations from different ecosystems and should be used in the future to understand the relationship between the gut and bacterial packet microbiomes, the development of the kudzu bug adult and nymphs, and the role of capsules in host plant utilization.

From the above review of endosymbionts in the kudzu bug, there is a great deal of evidence supporting their importance in the insect’s developmental biology. For example, it is clear that the kudzu bug has developed both morphological, physiological and behavioral mechanisms in the adult stage to produce bacteria packets concurrently with oviposition. This aspect of the biology requires: (i) a mechanism for the propagation of bacteria; (ii) mechanisms for the physical production of the packet itself; and (iii) a process for adding the bacteria to the packet. Furthermore, the insect also had to evolve behavioral processes to produce and deposit the bacterial packets from the digestive system at the precise time of oviposition (in a highly regulated ratio of the number of bacterial packets to the number of eggs). Whether there is communication between the female genital tract and digestive system to accomplish this coordination or both processes are controlled by the central nervous system is not known. Since egg production is regulated by at least juvenile hormone and also neuropeptides, the role of these hormones on the midgut, bacterial propagation, digestive function and packet production must also be considered. Finally, the first kudzu bug neonate meal is the bacterial packets. One reason for this is their proximity to the insects at the time of the hatch. However, the insect eggs are directly deposited on the host plant surface. Therefore, the packet or the contents of the packet (bacteria and/or maternal gut material) must be highly attractive and/or contain a feeding stimulus for the neonates since it is preferred over the readily available host plant material. This complex set of interactions between gut bacteria, the insect and the host plant clearly required co-evolution between all three organisms, which is only minimally understood.

## 10. Methods for Monitoring Kudzu Bug Population

**Figure 5:**
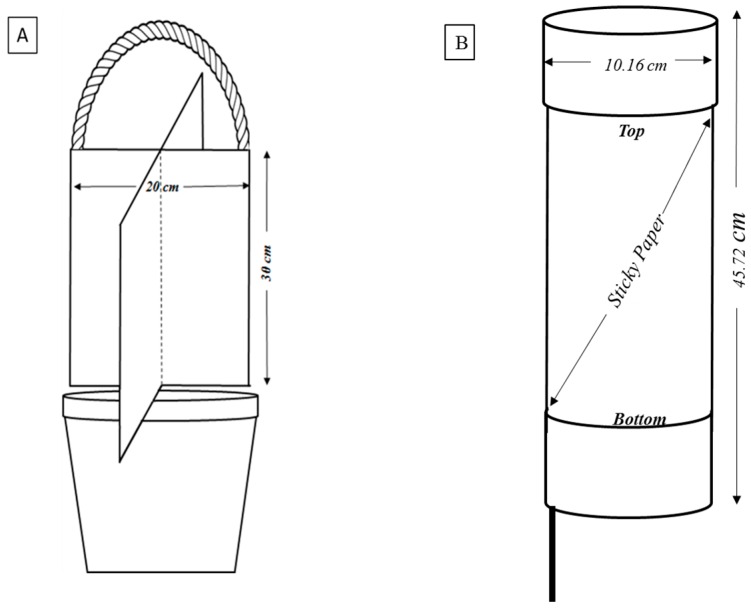
(**A**) Schematic diagram of trap used to collect kudzu bug by Horn and Hanula [31] which is based on Ulyshen and Hanula [59] with some modifications; and (**B**) flight capture trap used to study the emergence of kudzu bugs in Virginia.

Sampling of field populations of the kudzu bug has been conducted using cross-vane traps [3,31]; sweep nets and beat cloths [15,32]; and white polyvinyl chloride (PVC) adult sticky traps (described in more detail later in this section). In kudzu plots in Georgia, Zhang et al. [3] used cross-vane traps, proposed by Horn and Hanula [31], to capture and count adults. These cross-vane traps were a modification of a trap used by Ulyshen and Hanula [59] (Figure 5A). Cross-vanes that make up the trap were constructed by cutting grooves midway down the center of two plastic sheets (20 × 30 cm) and then interlocking them creating a cross-shaped barrier. The top center of the trap was attached with a wire for hanging, and the bottom was wired to a bucket (diameter 16 cm, depth 15 cm) [59]. Horn and Hanula [31] investigated the effect of the color on the effectiveness of trapping kudzu bugs. Out of the five colors (white, yellow, red, purple and black), white was most effective for attracting and capturing kudzu bug adults followed by yellow.

Since *M. cribraria* are highly attracted to upright white colored silhouettes, a trap developed by M. Towes [60] was used in our studies to monitor the kudzu bug in Virginia. A reusable sticky trap was constructed from a 30 cm long by 10.2 cm diameter white PVC pipe with an end cap cemented to one end; the pipe was then covered with disposable stable fly sticky paper that could be easily replaced by slipping the sticky paper off the bottom of the trap; traps were suspended about 1.5 m above ground level by driving a 1.8 m long wooden stake or steel rebar (1.3 cm diameter) in the ground and sliding the PVC pipe on the rebar (Figure 5B).

Passive traps, such as the cross-vane trap or PVC sticky trap, capture flying adults without operator action. The trap count is a simple function of time in the field, kudzu bug flight activity, abundance and environmental conditions that might influence flight. One disadvantage of this approach is that insects can travel from a long distance and be collected, and the trap count may not necessarily represent the kudzu population in close proximity to the trap. Furthermore, these traps only monitor the adult stage, not immatures, and as mentioned earlier the trap could be affected by weather or even the physiological condition of the insect. Sweep nets and beat cloths represent an active approach of population monitoring and are often preferred since they collect both immature and adult kudzu bugs, provide a more localized assessment of population density, and the sampling results are less affected by environmental conditions and insect development as compared to other methods. Of the two methods, the sweep net is preferred in soybean [15,32], discussed in more detail later (Section 11). The disadvantage of the active survey method is its requirement of an operator in the field to obtain data, and there could be inter-sampler bias in sampling efficiency.

## 11. Management of Kudzu Bug in Soybean

The main tool for the management of kudzu bug populations in their invasive range is the use of synthetic insecticides [33,34]. In China, up to 85% of *M. cribraria* in soybean are controlled with pyrethroids (β-cypermethrin, deltamethrin and sumicidin) and organophosphate insecticides [3]. The kudzu bug also can be an urban nuisance because of its dispersion to structures in the Fall of the year. Seiter et al. [35] examined the residual efficacy of nine insecticides including pyrethroids, neo-nicotinoids, and oxadiazine in urban settings. Residual efficacy was evaluated on vinyl soffits, brick, painted and unfinished plywood and metal for 1–30 days. Pyrethroids and pyrethroid-neonicotinoid mixes were highly effective with 100% mortality in 24 h. Dinotefuran gave similar results on smooth surfaces such as metal and vinyl but had a decreased residual efficacy on porous brick and wood. Although the oxidiazine, indoxacarb, was less effective, it remained active on porous surfaces for a longer time than other insecticides.

The chemical control of the kudzu bug is critical to pest management, but surprisingly little published work is available on the toxicology of this pest. For example, so far there are no probit models on dose response, of the impact of insect developmental stage and host plant on toxicity, the impact of synergists, or the importance of other environmental factors like temperature on insecticide efficacy. The kudzu bug in the Southeast U.S. is interesting because the population was originally established from a small number of “founder” insects (discussed in Section 1 and Section 2). The impact of this “founder effect” on variations in population susceptibility to pesticides and how this might change with pesticide pressure and as the insect migrates in the U.S. and adapts to new host plants and bacterial exposure could be an excellent case study of invasive biology. Without dose-response data, it is impossible to develop a knowledge-based resistance management strategy of risk analysis for the kudzu bug. Like many cases in the past, we are currently using insecticides without the most basic of information about the potential for insecticide resistance.

Similar to other sucking pests, a standardized resistance monitoring tool is needed and a resistance management strategy developed before resistance becomes a problem with kudzu bugs. Because all of the more effective insecticides in controlling kudzu bugs are disruptive to beneficial arthropods, the earlier timing of treatments will significantly increase the probability of establishment of subsequent populations of podworms, *Helicoverpa zea* (Boddie) and *Heliothis virescens* (Fabricius), presently the top insect pests of soybean in the southeastern U.S. The recommended action economic threshold recently established for the kudzu bug in commercial soybean fields is five adults per plant (when plants have six or less trifoliates, regardless of plant stage), one nymph per sweep, or a visual canopy observation of nymphs easily found on main stems and/or leaf petioles throughout the field interior [30,42]. Although recent data indicate that a single spray at the beginning of pod formation (R3 stage) in soybean could effectively manage kudzu bug and therefore minimize the total number of sprays [42], there were clear cases in experimental fields where a spray was not needed. Additional research showed that soybean susceptible stages to kudzu bug injury are pod and seed development [43]. Hence, most University Extension entomologists still recommend sampling and spraying selected pesticides at one nymph per sweep.

Stubbins et al. [32] developed a sampling plan based on economic threshold recommendations in soybean optimized for sweep-net versus beat-cloth sampling. Fewer insects were required to estimate the population size by sweep-net as compared to beat-cloth. Sweep-net sampling was more cost effective when density was low but as the density increased, the beat-cloth method had a greater cost benefit ratio [32]. Seiter et al. [42] demonstrated that a threshold of one nymph or more per sweep for spraying insecticides was sufficient in protecting soybean yield from losses due to *M. cribraria* injury.

## 12. Impact of Cultural Practices on Kudzu Bug Population Density

In a series of replicated tests conducted in South and North Carolina and Georgia over 2 years and at 6 locations, the impact of planting dates, soybean maturity group and insecticide-protected vs. unprotected plots were compared. Maturity group inconsistently affected kudzu bug adult or nymph density while planting date and insecticide protection significantly influenced pest levels, with the earliest planting resulting in the highest levels of kudzu bugs [11]. While it is known that earlier plantings are generally more at risk for kudzu bug infestation [10,11], it is not cost effective to delay planting due to yield reductions caused by abiotic and biotic factors later in the season. The same applies for the cultural management tactics of changing tillage regimes. Although the kudzu bug is less attracted to fields under reduced tillage [19], it is generally not cost effective for growers to modify tillage practices for an insect that can easily be managed using an insecticide. Early-planted soybean as a border around later-planted soybean fields have also been investigated as a trap crop. Although kudzu bug was initially more prevalent in the trap crop border than the field interior, field interiors surrounded by the trap crop became infested in equal levels compared to those without a trap crop border, indicating that further management of kudzu bug populations at the trap crop might be needed [61].

One promising management tactic under investigation other than insecticide use is host plant resistance in the form of antixenosis, antibiosis and tolerance. Several soybean genotypes have been identified with putative antixenosis and antibiosis [21,41]; the specific mechanisms of resistance are currently being investigated by characterizing trichomes on the leaves; and by collecting volatiles and free amino acids and identifying the compounds using HPLC. The hope is to then breed these traits into commercial germplasm that can be deployed in high yielding varieties for kudzu bug management.

## 13. Kudzu Bug Feeding Disruption Test (FDT) for Insecticide Susceptibility

**Figure 6:**
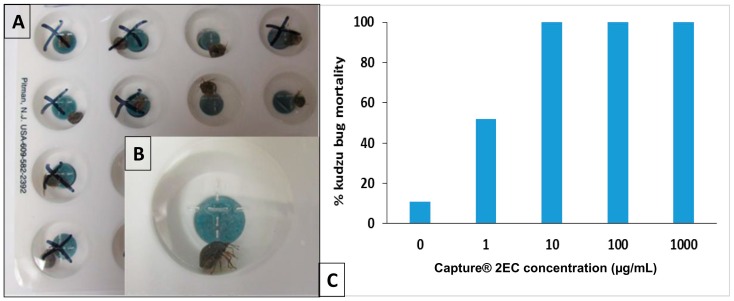
Feeding disruption bioassay for insecticide susceptibility of kudzu bugs: (**A**) 16-well plate and exposure results for a 24 h assay. Wells marked with an “X”, contained a dead insect; (**B**) Close up of a single well. The blue disc in the center of the well is a hydratable caterpillar diet. At the start of the assay, the meal pad was hydrated with an aqueous concentration of the insecticide Capture, a single kudzu bug adult added to the well, and the well covered with a ventilated, transparent plastic (self-sealing) lid; and (**C**) Dose–kudzu bug mortality response for the assay shown in **A**. The concentration of the insecticide shown is that found in the hydration solution, not in the final hydrated diet. Mortality was defined as no movement when the insect was touched with a blunt probe and/or did not move when the plate was agitated. There was no control (no insecticide) mortality observed.

To manage the use of insecticides for insect control, it is useful to have a bioassay system to evaluate insecticide susceptibility. Feeding disruption test (FDT) is a diagnostic method developed for a variety of insecticides, including chemical insecticides, Bt, other protein toxins and RNAi-based pesticide technologies and for a variety of insect pests from different insect orders and with different feeding mechanisms [62,63,64,65,66,67,68,69,70,71,72,73,74,75,76,77,78,79]. The kits consist of a specially designed white plastic 16-well plate with recessed, hydratable meal pads, which are mass-produced robotically. The meal pads contain a diagnostic dose of insecticide and a blue indicator dye to monitor larval feeding. The appearance of blue feces (a measure of feeding rate) can easily be seen on the background of the white plate after a 4–24 h incubation period in resistant insects. The absence of blue feces occurs because the susceptible insects are intoxicated by the insecticide and do not feed. Mortality can also be used as a bioassay endpoint. Figure 6A,B shows a working system for kudzu bugs using FDT with a typical dose-mortality response (Figure 6C). In this case, the insect is exposed to the insecticide by obtaining moisture from the diet and when crawling over the diet surface but did not find the food palatable. Future work is needed to develop and use an artificial kudzu bug diet if a fecal production end point is desired.

## 14. Kudzu Bug Natural Enemies

Rapid expansion of the kudzu bug in the initial years following its introduction into the U.S. has been followed by rapid slowing of its expansion in recent years (Figure 1) [6]. Furthermore, in areas of its first establishment, the kudzu bug pest status has declined. For example, in North Carolina, it was initially found on soybean in 2011. Percent estimated soybean hectares infested were 1.1, 21.9, 25.4, 28.4, and 25 during 2011–2015, respectively, with an estimated 0.7%, 3.6%, 12.3%, 3.0%, and 0.001% hectares sprayed during 2011–2015 [80,81,82,83]. Whether these trends will continue is unknown. There are several possible reasons for the decline including weather related mortality (for example colder winters), predators, parasitoids, and diseases (naturally occurring epizootic episodes of *Beauveria bassiana* in experimental/commercial soybean fields), which are presently unquantified.

Some research is available on biological control of the kudzu bug. There are several known adult parasitoids, two tachinid flies, *Phasia robertsonii* (Townsend) [12] and *Strongygaster triangulifera* (Loew) [36], and a mermithid nematode [84] that use the kudzu bug as a host. Garner et al. [37] surveyed for egg parasitoids in discolored egg masses collected from kudzu at the University of Georgia (Griffin, GA, USA) and found a high parasitism rate by the wasp, *Paratelenomus saccharalis*. Recently, this parasitoid was also reported in Florida by Medal et al. [38]. This wasp is an egg parasitoid found in the eastern hemisphere and more recently in the U.S. and typically involved in parasitism of *M. cribraria*, *M. punctatissimum* and *Brachyplatys subaeneus*. Finding this parasitoid in the U.S. and associated with the kudzu bug suggests that the parasitoid was most likely introduced into the U.S. in *M. cribraria* eggs. It seems less likely there was a simultaneous but independent introduction of both the adult wasp and egg laying kudzu adults or unparastized eggs at the same time and place. The recent discovery of this wasp in the U.S. also suggests that the introduction of the kudzu bug may also have been at the egg stage. Zhang et al. [3] observed no egg parasitoids when they examined *M. cribraria* eggs collected from kudzu vines in Georgia.

**Figure 7:**
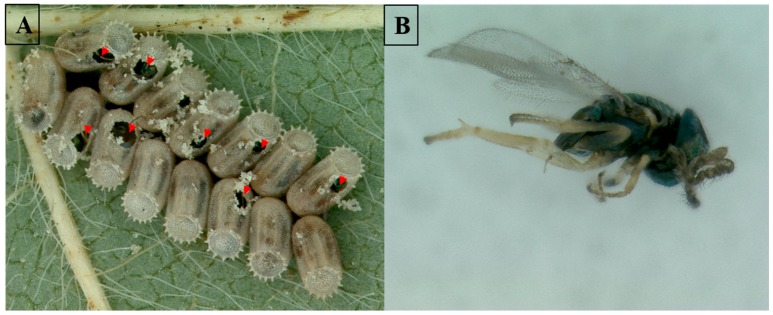
*Ooencyrtus* sp. wasp discovered as an egg parasitoid of the kudzu bug in Virginia: (**A**) Kudzu bug egg mass with parasitoid present (red arrows indicate presence of the parasitoid); and (**B**) adult *Ooencyrtus* sp. wasp that emerged from the egg mass.

We recently surveyed kudzu bug eggs for parasitoids in North Carolina and Virginia. Eggs were collected from either kudzu or soybean and transferred to the laboratory for observation in 2014 and 2015. In 2014, 2596 and 1982 eggs were collected from North Carolina and Virginia, respectively. No parasitoids were observed from eggs collected in North Carolina but two potential parasitoids emerged from eggs from Virginia, egg parasitic wasps from the genera *Gonatocerus* and *Eretmocerus*. Wasps from the genus *Gonatocerus* have a broad host range while *Eretmocerus* wasps are more specialized with known parasitism of white flies. Although care was taken in these studies to collect only kudzu bug eggs, there is always the possibility of contamination from eggs of other insects which could lead to false positives. In the survey conducted in Virginia in 2015, 11,205 eggs were collected and three potential parasitoids were found in the genera *Gonatocerus*, *Encarsia* and *Ooencyrtus*. *Gonatocerus* and *Encarsia* were found in the rearing arena and were assumed to have emerged from kudzu bug eggs as was the case for the egg parasitoids found in 2014. The *Ooencyrtus* sp. wasp was discovered in the kudzu bug eggs (Figure 7A,B) leaving no question that they were feeding on the eggs.

In July 2013, Golec et al. [36] collected adult kudzu bugs from soybean fields in Auburn (Lee Co., Auburn, AL, USA) using beat-cloths. From 214 bugs collected, 5.14% were parasitized with females having a higher rate of parasitism. The parasitoid was identified as *Strongygaster triangulifera*, which is a parasitic fly widely distributed in North America. *S. triangulifera* is a generalist parasitoid and usually parasitizes Coleoptera in more than 10 host families [36].

Greenstone et al. [39] investigated the predation of kudzu bugs in soybean neighboring a cotton crop system. This study used molecular gut content analysis to identify the food consumption of general insect and spider predators. This study not only investigated the predation of kudzu bugs but also examined the migration of these predators between crop systems. Eight native predators were found feeding on kudzu bugs (two geocorids, one anthocorid, one pentatomid, one coccinellid, one reduviid and two oxyopid spiders). A single exotic predator was feeding on kudzu bugs, *Solenopsis invicta* Buren. To understand the migration of these predators, DNA gut analyses were conducted for three additional insects (*Piezodorus guildinii* and *Thyanta custator* found only in soybean and *Euschistus tristigmus* found in cotton in this study). Combinations of kudzu bug with *P. guildinii* and *T. custator* and combinations of kudzu bug and *E. tristigmus* were found in cotton and soybean, respectively, suggesting predator movement between these two crops. These findings argue that the close proximity of cotton to soybean could enhance kudzu bug biological control.

Seiter et al. [40] confirmed by purification and inoculation, that kudzu bug adults can be infected by *Beauvaria bassiana* clade A at a noticeable, apparently density-dependent mortality level in an artificially confined population of *M. cribraria*. Further studies should examine the pathogenicity of diverse strains of *B. bassiana* as well as evaluate commercial formulations for organic management of *M. cribraria* [40].

## 15. Future Directions

The introduction of the kudzu bug into the U.S. is not only the introduction of a new insect species but a new family of insects not seen before on this continent. This is a “once in a life time opportunity” to study the evolution of an invasive organism and the dynamics of its integration into the U.S. ecosystem where the insect has unique biological features. Its special association with bacteria is one of these unique features and represents the co-evolution of an insect with obvious specialized physiological and behavior mechanisms to maintain this relationship with not so obvious adaptations in its bacterial community. Understanding this co-dependence will be informative both in advancing our understanding of the association of animals with bacteria but will also likely provide leads on new methods for kudzu bug and insect control. Additionally, there is the likely possibility that the introduction of kudzu bug eggs into the U.S. occurred along with the co-introduction of a new parasitoid into the North American continent and with it a unique community of bacteria and other microbes for both insects. How this impacts other insects is unknown.

Studies are greatly lacking in understanding the potential development of insecticide resistance management programs, and understanding the role of beneficial arthropods, pathogens and other organisms in kudzu bug population dynamics. The deployment of insecticides and understanding migration between wild hosts and crop plants like soybean will be essential to develop a sustainable IPM program, avoiding the sole reliance on pesticides, in addition to evaluating the potential for the kudzu bug to move to other crops like fruits and cotton. The impact of this insect, including its microbiome and chemical defenses, on urban systems including humans and animals so far appears to be minimal but should be further assessed. Finally, there are questions about the species identification of the kudzu bug in the U.S., which need further study. The introduction of the kudzu bug into the U.S. has serious negative practical implications but paradoxically, also provides an opportunity for scientific studies with potential future benefits.

## Figures and Tables

**Table 1:** Prominent areas of kudzu bug, *Megacopta cribraria*, research.

Research Area	Topic	References
Biology	Morphology	[1,3,13]
	Life cycle	[1,3,4,13,14,15,16,17]
	Spatial distribution	In plants: [18,19]
		In the field: [20]
	Population dynamics	[9,20,21]
	Overwintering	[12,22]
	Identification in US	[1,23,24]
Host and habitat	Native range	[1,25]
	Discovery and prevalence in the U.S.	[2,3,4,5,13,15]
	Host range and preference	[1,3,14,16,26,27,28,29]
	Impact as pest	[1,9,12,13,30]
Control and Management	Trap/sampling	[15,26,31,32]
	Chemical control	[3,30,33,34,35]
	Natural enemies	[3,36,37,38,39,40]
	Cultural management	[10,11,19,21,41]
	Thresholds	[42]
	Based on soybean phenology	[43]
Microbial Interaction	Identification and role of symbionts	[44,45,46]
	Transfer of symbionts	[44]
	Host selection	[14,28,44,47,48]
	Evolution	[25,44]
